# Florence Nightingale

**Published:** 1889-07-27

**Authors:** 


### Florence Nightingale.

There's naught in this bad world like sympathy;
'Tis so becoming to the soul and face.
Sets to soft music the harmonious sigh,
And robes sweet friendship in a Brussels lace.
—*D. J. Byron.*

Unfee'd, the calls of Nature she obeys;
Not led by profit, nor allured by praise.
– *Crabbe.*

The name of Florence Nightingale is probably more widely known than that of any other woman who lives, or has lived. There is scarce a country, be it in Europe, Asia, America, or in the Southern Hemisphere, in which the tale is not told of this lady's heroic devotion to a noble and self-imposed duty. And evidence is not wanting that the world has gained largely by her work and example.

Though known to the great mass of her countrymen chiefly through her campaign of nursing and hospital work in the Crimea, Miss Nightingale's whole life has been one of continuous and persistent devotion to benevolence and to ameliorating the condition of her fellow-man. She was born in May, 1820, in Florence. The bright Italian sky, and the fairest city of an ancient land over which it smiled, were auspicious surroundings enough; and much of the beauty and perfection of the godmother is reflected in the loving and assiduous labours, the life's sacrifice, the wide and genuine sympathy of the woman who bears her name.

Miss Nightingale enjoyed all the advantages which fall to the lot of the children of the affluent and refined, her father, Mr. W. E. Nightingale, a very cultivated and philanthropic man, himself superintended the education of his two daughters. At Embley Park, Hampshire, and at Lea Hurst, Derbyshire, her earliest days were spent among the poor, visiting, helping, and cheering them. Then, as the mind matured, it widened the radius of its operations, and the actual working of every kind of institution in England bearing upon the promotion of moral and physical happiness became an object of Miss Nightingale's investigation. Hospitals, schools, and reformatories were her study, and whilst other young women followed the usual course, and allowed themselves to be carried away by the social pleasures of the day, she devoted herself untiringly to this work of practical philanthropy.

The instinct which had made Miss Nightingale not content to continue at local country work in the neighbourhood of her father's estates, and had induced her to examine the condition of all our British institutions, was not to be satisfied even thus. During the year of the great Exhibition, in 1851, she spent some months with the Protestant Deaconesses at Kaiserwerth, and at Paris among the Sisters of Mercy, conducting enquiries similar to those she had commenced in England, and extending her researches over a vast and important field. But she did not forget her country, for it was shortly afterwards that the Sanatorium in Harley-street, London, was re-organised and re-established solely by Miss Nightingale's energy and liberality.

But such experiences as these, good and beneficial as they were, were trifles when compared with the great work which it was shortly to be her lot to undertake. The years slowly rolled on, until the terrible 1854 was present. Miss Nightingale was now thirty-four. Thousands of British soldiers were fighting in Eastern Europe. Far from home, ill-provided with necessaries, and oppressed with a climate of terrible extremes, they were victims at once of war, pestilence, and hardship. Rumours reached England of the inefficiency and mismanagement of our military hospitals in the Crimea, and the sympathy of every class was at once aroused.

Alma was just won, and the nation was jubilant, but Sir Robert Peel wrote in the *Times* that the men who were winning all this glory were martyrs to much unnecessary suffering. A Patriotic Fund was established, and money poured in—£7,000 in the first seven days, and £25,462 altogether. It was decided to send a band of trained women out into the thick of all the sickness and carnage, and so alleviate, if possible, the sufferings of the wounded. It seems natural now that Miss Nightingale should have been selected to lead this little band of thirty-seven devoted women. She was eminently fitted for the duty, both because of her practical knowledge, her experience, and her powers of organisation and direction.

It was in November of 1854 that Miss Nightingale disembarked at Constantinople with eight Protestant sisters, six nurses from St. John's Institution, six Catholic nurses, and the rest selected from various hospitals in England. She immediately established herself at the Scutari Barrack Hospital, and was just in time to take charge of the wounded as they arrived from Inkerman.

The fame of the little band of nurses had preceded them. All Europe watched them and applauded. In France, the hotel-keepers refused payment, the servants their fees, the highest joining with the humblest in paying tribute to these devoted women, and showing their appreciation of their work.

Passing through Boulogne, the fishwives insisted upon carrying their baggage; and a French newspaper published the following panegyric: "Miss Nightingale possesses all that could render existence happy and brilliant. Young, handsome, and wealthy, she has chosen a life of abnegation and self-denial."

It was long before the chaotic confusion which had existed in the vast hospital at Scutari was reduced to order, or relief given to the tremendous mass of suffering there. The barrack hospital at Scutari was three storeys high; every corridor and every room was full of patients; it was calculated that there were four miles of beds set nearly as close as they could stand. In seventeen days, at the end of December and beginning of January, 1855, nearly 4,000 patients arrived from the front. But the feeling that there was someone at hand to do what was possible, and that England had thus shown that she felt and cared for them, comforted many a poor fellow, who, as they said "could kiss her shadow as it fell," in her perpetual progress through those miles of misery.

As the nation read eagerly the accounts of the battles, and anxiously followed the progress of the war, so did it also take note of the success that attended Miss Nightingale's work. "Her devotion to an enterprise so painful, so arduous," we read, "had ensured her the enthusiastic applause of her countrymen; and their favour became part of her strength."

There was something so dignified, so resigned, and yet so sympathetic in Miss Nightingale's bearing that her presence beside a sick-bed encouraged and strengthened the sufferer. Men who were broken and almost unmanned with pain and disease, and who shrank from the terrors of the operating table, faced it with fortitude when they realised that this gentle being would stand silently beside them, sharing their sufferings and going through extremes of mental pain, caused by an overflow of sympathy which was real and unmistakable.

A nurse wrote from the hospital at Scutari: "I don't know which sight is most heart-rending to witness; fine, strong men and youths, worn down by exhaustion, and sinking under it, or others coming in, as many hundreds did yesterday, fearfully wounded." These were the scenes and painful experiences which had to be faced in the great nursing campaign which Miss Nightingale undertook.

Of her method and strictness of organisation too much cannot be said. It is a rare combination of virtues when ntelligence and will, deliberation and judgment, are found side by side with modesty and reticence, kindness and womanly sympathy. In Miss Nightingale, all these qualities were united. "With all the rare attributes that made her gracious presence a blessing," writes Kinglake, "at the patient's bedside, this gifted woman, when learning how best to compass the objects of a largely extended benevolence, had become well-versed, well-practised in the business of hospital management; and none knew better than she did that if kind, devoted attention will suffice to comfort one sufferer, or even perhaps four or five, it is powerless to benefit those who number by thousands, unless reinforced by method, by organisation, by discipline."

So much faith, indeed, did she have in rules, and so deep a sense of the value of methodical arrangement, that it was feared by many that she would become too strict rather than too lax. "The officials at Scutari," we are told, "were not slow to perceive that the lady in charge of the nurses had laid a firm hand on a lever, which, against all objection, and even against sheer inertness, was enforcing good hospital management."

This characteristic is extremely noticeable in all Miss Nightingale's writings, and in all her work. But it is everywhere and at all times blended with the softer side of her nature, and so perfectly that neither unduly preponderates. She is described as slender and delicate in form, engaging, and highly bred. In council, a rapt and careful listener so long as others are speaking, and strongly, though gently, persuasive whenever speaking herself. Her forethought is accompanied and strengthened by an active brain-power, by an "organising and governing faculty." She possesses the intuition of a woman, and can utilise it to an extent unusual to her sex; she has that subtle influence which gives to one human being a command over others. To these qualities Kinglake adds "soundness of judgment on questions needing rapid decision, and an apt and ready knowledge, with which she always seems armed." In situations of difficulty and danger Miss Nightingale has also shown a "keen discrimination, enabling her to judge, at the instant, whether any of the words addressed to her should be treasured or set at nought." She was persistent and undaunted by reverses, and from her early years "has been impelled by an impassioned benevolence to devote great power of mind and unconquerable energy" to the objects into which she has so completely thrown her life.

Another description of her personal appearance in 1854 is appended, and is taken from a letter written by a gentleman at Scutari at the time: "In person she is tall and graceful, her head is small and finely shaped, her face full of feeling, sense, and humour, her eyes have the charm of the most womanly softness, and the upper lip and mouth display unlimited courage. What has she not carried out?"

"Her gift," writes the admiring Crimean historian, "was her faculty of conquering dominion over the minds of men," and "this was the force which lifted her from out of the ranks of those who are only 'able,' to the height reached by those who are 'great.' One who would not, I know, be prone to misuse our most choice words of praise, has ascribed to the Lady-in-Chief nothing less than 'Commanding Genius.'"

From quite another source did the following eulogy emanate, but it still further exemplifies the combination of character which has already been referred to: "Few know of the heroic conduct of Miss Nightingale in the hospital at Scutari. Not only has she since her arrival attended the death-beds of the soldiers under her charge, but has had the most dangerous cases placed in the corridor and rooms next to her own, that she may be near, and thus able to render greater assistance."

These estimates of character may be accepted as accurate and reliable, made, as they are, by those who have enjoyed the personal acquaintance and friendship of Miss Nightingale, and seen her at her work. It is in moments of anxiety, such as at the still watch at a death-bed, or in moments of trial, as when the frail frame is overcome with weariness, and the spirit oppressed with the continued sombreness of its surroundings, that a character really displays itself. And it was in such moments that the world saw the power, the originality, the ready tact, and the womanly goodness of Miss Nightingale.

It is true that Miss Nightingale lives now in retirement, but she is working as hard as ever for hospitals all over the world, for the moral and physical welfare of the soldier, and for the help of the vast Hindoo population, especially the women.

"Every woman," she writes in her characteristic "Notes on Nursing," "or at least almost every woman in England, has at one time or another of her life charge of the personal health of somebody, whether child or invalid. In other words, every woman is a nurse. . . . How valuable, then. . . would be the produce of their united experience if every woman would think how to nurse."

Following up the thread of this philosophy, she discusses the increasing tendency to clamour for special institutions for the alleviation of sickness and suffering. She admits, of course, the necessity for such institutions, but lays great stress upon the obvious duty of all to help themselves. Household and motherly care are, to her, even more important than hospitals for women and children. She realises to the fullest extent what woman's natural sphere is—in what manner she can best direct her energies—how, in fact, she may most benefit her fellow-creatures.

In all the force of character, the decision which over-ruled and even awed the officials at Scutari, the self-reliance which prompted her to lead an expedition into a foreign country and amidst perils of every kind, there is nothing in Miss Nightingale that offends the highest ideal of woman, working in her own peculiar sphere, softening, refining, and influencing for good all whom she comes in contact. There is nothing unduly imperious in her genius, "commanding" though it be. There was no harshness in her love of methodical arrangement, though it was strict and practical when occasion needed. She is deliberate and critical, but her private fortune and personal labour have been dedicated to a single, noble, and philanthropic object.

Miss Nightingale was absent from England for a short two years, and in that brief space of time had risen into fame. She had suffered once from Crimean fever, and although she recovered, the attack is supposed to have laid the seeds of her subsequent ill-health. Indeed, it is sad to think that ever since her return from the short, but brilliant, exploit in the Crimea, Miss Nightingale has been an invalid; for many years she has been almost entirely confined to her room, seeing only those who could help her in her work. The English people, who are never slow to recognise and reward merit, subscribed the magnificent testimonial of £50,000 on her return; but the money was, at her request, devoted to the formation and maintenance of an institution for the training and employment of nurses at St. Thomas's Hospital.

A public meeting was held in Willis' Rooms, the Duke of Cambridge presiding, when it was resolved unanimously: "That the noble exertions of Miss Nightingale and her associates in the hospital, and the invaluable services rendered by them to the sick and wounded of the British forces, demand the grateful recognition of the British people; that it is desirable to perpetuate the memory of Miss Nightingale's signal devotion, and to record the gratitude of the nation by a testimonial of a substantial character; and that as she has expressed her unwillingness to accept any tribute designed for her own personal advantage, funds be raised to enable her to establish an institution for the training, sustenance, and protection of nurses and hospital attendants."

The reticence and modesty of this great woman are evident in the following paragraph, also taken from her writings: "We do not seek to make medical women, but simply nurses acquainted with the principles which they are required constantly to apply at the bedside."

The effect of Miss Nightingale's work and example in England, in America, and, indeed, all over the world, is already apparent. "By the power of her fame, by the wisdom and authority of her counsels, she founded, if one may so speak, a gracious dynasty, that still reigns supreme in the wards where sufferers lie, and even brings solace, brings guidance, brings hope, into those dens of misery that, until the blessing has reached them, seem only to harbour despair."

Every great city now has its little army of self-sacrificing and devoted women, who undergo a somewhat severe course of training, suffer the pain which the sight even of pain inflicts, eschew the empty and flaunting pleasures with which our social life, unhappily, abounds, and spend their days in trying to ameliorate the moral condition and alleviate the physical sufferings of others. The example of a refined woman, a wealthy woman, a clever woman, throwing herself into this kind of work, has proved contagious; and to-day we find, what previous generations were entirely without, gentle manners, high moral tone, refinement, education, and ability, hovering around the sick-bed, hallowing the chamber of death, softening and cheering the very atmosphere of the solemn hospital ward.

This is but one result of Miss Nightingale's work, and it is the softer, the more humane side of it. Another, and a practical effect, is that a new calling has been established into which respectable women of every class may enter and earn a living; and so rapid are the strides which the times are making that nursing is fast being raised to the dignity of an art, its members being recruited from all ranks of society. It is a mistake to suppose, and we are far from encouraging the idea, that the reform which has followed upon Miss Nightingale's work is due to a substitution of ladies for the ordinary nurse of the humble classes. The public, who know little of the hospitals, are, however, very apt to run away with this idea. The reform really lay in the introduction of an organised system where none previously existed; in putting trained and educated women at the head of all nurse-training and other institutions; in conferring upon them due authority over the nursing staff; providing the means for proper discipline, efficient instruction, and good accommodation. All these innovations, together, have tended to make nursing what it is—a respectable and worthy calling; and to Miss Nightingale's initiative much of the substantial reform which has been realised is due.

Who shall estimate the value of such an example? Who reward the lady, who has stimulated her countrywomen to do such good works? Money, she does not need; has actually refused; fame, though it has settled firmly upon her, has never been courted. But all English-speaking people have a right to admire and appreciate worth, and they do so.

The writer was once in a distant land, where English live, far from their home, but always fervent in their affection for it. An American, a colonel who had served with some distinction in the civil wars, announced a lecture on "Famous Women." The large theatre was packed, and one by one the speaker sketched, with great eloquence, the lives of women who had earned fame. At last he referred to a great war in Europe; to the sufferings of hundreds of wounded and their dreary surroundings as death closed upon them He whispered, rather than spoke, to a thousand eager listeners, how a woman's form had flitted about the camps cheering, aiding, soothing, sympathising with the sufferers. He told of the devotion, the noble unselfishness, the increasing and racking labours of one who had risen above the level of her sex; and when he reached the climax and named Florence Nightingale, the enthusiasm of the audience burst out, and they thundered forth their applause. Not a hand was still, scarcely an eye dry, as the memory of these deeds was aroused. For even in that distant land the history of Florence Nightingale's achievements is reverenced and appreciated. "The lady of the lamp," as she is termed in Longfellow's beautiful poem.

Her life's work has been performed with a singular absence of display, and she has never for a moment courted publicity or fame. Yet the name of Florence Nightingale has been uttered in almost every corner of the earth, and her deeds of devotion and noble unselfishness have already influenced the daily lives of many thousands of women of the present day.

Much of Miss Nightingale's work has been done so unostentatiously, that its nature and extent are not known to the great mass of the English people. The valuable assistance she rendered to Lord Herbert, when head of the War Office, in framing those measures of sanitary reform by which the health of the soldier has been so greatly improved. Her work in India for the sanitation of the barracks and stations all over the country. At the time of the Franco-German and American wars she was consulted about the care of the sick and wounded, plans for hospitals and for the improvement of nursing coming to her from all parts at home and abroad.

Among other writings, Miss Nightingale has published "Notes on Hospitals," which appeared in 1852, and had a very large circulation; "Notes on Nursing," 1860, of which nearly a hundred thousand copies have been sold; and many papers sent to the British Association and published in different reviews.

